# Life expectancy in patients with degenerative cervical myelopathy is currently reduced but can be restored with timely treatment

**DOI:** 10.1007/s00701-023-05515-8

**Published:** 2023-03-01

**Authors:** Benjamin M. Davies, Daniel Stubbs, Conor S. Gillespie, Ben Grodzinski, Ashwin Venkatesh, Matthew Guilfoyle, Mark R. N. Kotter, Rodney Laing

**Affiliations:** 1 Department of Clinical Neurosurgery, University of Cambridge, Cambridge, UK; 2 University Department of Anaesthesiae, University of Cambridge, Cambridge, UK; 3 Myelopathy.Org, Cambridge, UK

**Keywords:** Cervical myelopathy, Cervical Spondylosis, Cervical Stenosis, Ossification of the posterior longitudinal ligament, Degeneration, Survival, Degenerative Cervical Myelopathy

## Abstract

**Purpose:**

Degenerative cervical myelopathy is a progressive slow-motion spinal cord injury. Surgery is the mainstay of treatment. Baseline disability predicts surgical recovery; therefore, timely treatment is critical to restoring function. However, current challenges mean most patients present with advanced disease and are instead left with life changing disabilities. While short-term mortality is rarely reported, the long-term effects of this on life expectancy are unknown, including whether function could be modifiable with timely treatment. This article investigates the effect of DCM on life expectancy.

**Methods:**

The survival of patients from an observational study of patients undergoing surgery from 1994 to 2007 was compared to their expected survival using a gender- and aged -matched cohort. Comparisons were made by one sample log-rank test and standardised mortality ratios. Factors associated with survival were explored using a Cox regression analysis, including disease severity.

**Results:**

A total of 357 patients were included in the analysis. After a median follow-up of 15.3 years, 135 of 349 patients had died; 114.7 deaths would have been expected. The DCM cohort had an increased risk of death compared to the non-DCM cohort (standardised mortality ratio 1.18 [95% CI: 1.02–1.34]. Age at operation 1.08 (95% CI: 1.07 to 1.1, *p* < 0.001) and severe DCM 1.6 (95% CI: 1.06 to 2.3, *p* = 0.02) were associated with worse survival (*N* = 287). In those surviving at least 2 years after surgery, only severe DCM was associated with conditional survival (HR 1.6, 95% CI 1.04 2.4, *p* = 0.03).

**Conclusion:**

Life expectancy is reduced in those undergoing surgery for DCM. This is driven by premature mortality among those left with severe disability. As disability can be reduced with timely treatment, these findings reinforce the need for collective and global action to raise awareness of DCM and enable early diagnosis.

**Supplementary Information:**

The online version contains supplementary material available at 10.1007/s00701-023-05515-8.

## Introduction

Degenerative cervical myelopathy (DCM) is a form of slow motion and evolving spinal cord injury [20]. It is estimated to affect 1 in 50 adults [66], although today most are never diagnosed. [35, 63] In DCM, spinal cord damage is driven by mechanical stress brought about by degenerative/spondylotic changes to the spine, such as disc prolapse, spondylolisthesis, ligament hypertrophy, or ossification [7]. This can cause a variety of different symptoms [24, 57] and experiences. [44] While changes in dexterity, gait, bladder function, limb pain, and sensation are the focus of current assessments [18, 23, 40], this under represents a disease, which can affect the whole body, from internal organs such as the cardiorespiratory system [11, 13, 47, 48, 74] to perhaps even cranial nerves [14, 15, 24, 54]. This very individual experience of DCM is governed by a likely complex and poorly understand interaction between mechanical stress, time, and an individual’s vulnerability to spinal cord injury [26].

Surgery to remove the mechanical stress on the spinal cord is the mainstay of treatment[31]. It has been shown to stop further spinal cord damage and enable recovery [30]. However, due to the limited capacity of the spinal cord to repair, a ‘full’ recovery is therefore dependent on offering surgery before there is irreversible damage. [68] Today, most patients reach surgeons with advanced disease after years of symptoms. [62] Based on healthcare activity in the UK, it has been recently estimated that ~ 90% of DCM may go undiagnosed, particularly among the elderly [35]. Therefore today, recovery is generally incomplete. Instead, most patients are left with life-long disabilities associated with high levels of dependence and unemployment [62]. A recent report by the DCM charity, Myelopathy.org, has estimated this equates to an average lifetime loss of earnings of ~ £0.5 m for those of working age, with a conservative cost to English society of £0.7bn per year [25].

In the short-term, at least, mortality is rarely reported [18], even among those undergoing surgery [75] but for a group left with multisystem disability, is this true life-long? While some surgical series on DCM have reported longer-term outcomes[3, 31, 59] [4, 33], these rarely stretch beyond 5 years. Moreover the wider implications of a life with DCM are unknown. Further, if we can offer surgery in early stages of the disease, might any such impact be modifiable? In this study, we examine for the first time the effect of DCM on life expectancy.

## Methods

### Study design and patient population

This was a case–control study. The observed survival of a series of adult patients (> 18 years) who underwent surgical treatment for DCM and were enrolled to a prospective observational study from 1995 to 2007 [4, 46], was compared to their expected survival using a matched cohort of the general population, generated and matched for gender and year of birth using data from the Office for National Statistics, UK [1]. Survival status was retrieved following ethical approval and patient consent on August 1, 2020 using the National Health Service (UK) Spine. We included patients in the DCM surgery group if: were adults enrolled during the study who had follow-up of a minimum of 6 months post-operatively. Patients were excluded if they had missing survival data or if they did not complete the study. This includes the survival status of the UK population accurate to within 6 months.

### Outcomes assessed

The following variables were obtained: age, sex, physical functioning domain of the SF36v1 and the Myelopathy Disability Index [MDI], survival at time of search query, and length of follow-up. Disease severity was measured using the MDI and its criteria for mild, moderate, and severe disability. [17]

### Statistical analysis

Statistical analysis was performed in R (v3.60 www.r- project.org) using the ‘survival’ and ‘relsurv’packages. [60, 61, 73] To explore survival, a comparable cohort in terms of age and gender was created using annual age- (in 1-year increments) and sex-specific risk of death from the human mortality database for England and Wales [36]. Comparisons between observed and expected survival were then made using Kaplan–Meier survival methods, with a one-sample log-rank test [12] and standardised mortality ratios (SMR) used to detect the difference between groups. Significance was assessed using a chi-squared test on one degree of freedom as per previously published methods. [67]

Variables associated with survival were explored individually and in combination using a Cox regression [38]. Regression co-variates were selected using backward stepwise elimination, based on improving the Akaike Information Criterion (AIC). Alongside age and gender, the co-variates of interest were pre-chosen from the available postoperative outcome measures to represent health status after surgical treatment. Specifically, these were the physical functioning domain of the SF36v1 and the Myelopathy Disability Index [MDI]. Aside from gender, these were therefore continuous or ordinal variables and were categorised for analysis. The MDI was categorised as mild, moderate, or severe based on predefined thresholds [17]. The SF36v1 Physical Functioning Component Score dichotomised as either having achieved the minimal clinically important difference [MCID] or not[6]. The SF36v1 was chosen for this purpose, principally as such a threshold for the Myelopathy Disability Index has not been ascertained [79] and MDI a measure of DCM severity. The best value of outcomes from 12 to 24 months was taken to coincide with the window of peak recovery from surgery and minimise missing data[59]. Analysis of co-variates and regression testing was restricted to complete cases. Statistical significance was set at 5%. This analysis was then repeated using only those who had survived at least 2 years after surgery, referred to as the conditional cohort or conditional survival [61, 67]. This was performed to strengthen the identification of factors that influenced long-term survival.

## Results

### Study population

Cohort demographics are given in Table 1. A total of 357 patients were included in the DCM cohort. The mean age of the cohort at surgery was 55.8 ± 14.9, and 196 (56%) were male. The disease and treatment characteristics have been reported elsewhere [24, 31, 42], but in short, the original observational study was compiled by the senior author (RJCL), and we included patients with a clinical and imaging diagnosis of DCM who underwent surgical treatment. Patients completed patient-reported outcome measures preoperatively and at 3, 12, 24, and 60 months postoperatively. Outcome measures included the SF-36 (version 1) quality of life measure, the visual analogue scores for arm, neck, and hand pain, and condition-specific outcome measures the Myelopathy Disability Index [MDI] and the Neck Disability Score [NDI]. Most patients were treated via an anterior approach (ACDF- 247, 70.2%), with all others treated with a posterior approach. The median follow-up was 15.3 years after surgery (IQR 7.5, range 0.3–24.9).

**Table 1:** Cohort demographics

Variable	Value
Age at operation (± SD)	55.9 (± 14.9)
Male gender (%)	195 (56)
MDI* (%) Mild Moderate Severe	148 (52) 42 (15) 97 (33)
PCS* (± SD)	47.4 (32.4)
MCID achieved* (%)	152 (58)

*MDI*, Myelopathy Disability Index; *PCS*, physical component score; *MCID*, minimal clinically important difference

^*^ denotes variable with missing follow up data. For these variables, proportions are given for cohort with outcome data

### Life expectancy estimate

**Fig. 1:**
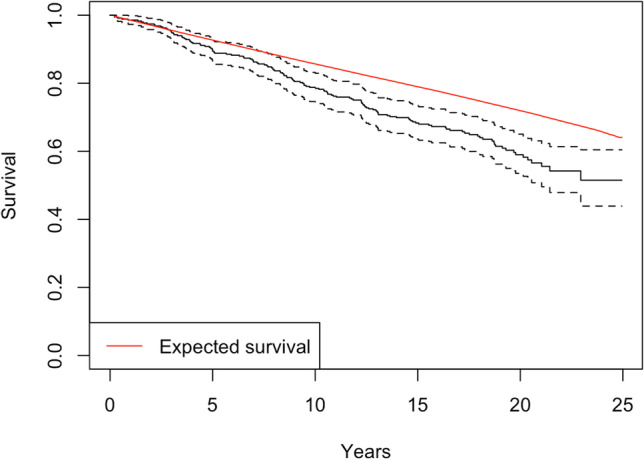
Observed and expected survival of cohort. The diagonal red line represents the expected survival for an age- and gender-matched cohort. The black curved line represents the observed survival, with 95% confident intervals (dotted line) for the cohort

One hundred thirty-five of 349 (38.7%) of patients died within the follow-up period. Average survival was 15.3 years (± interquartile range 7.5) and ranged from 0.3 to 24.9 years. From a corresponding age- and sex-matched sample, 114.7 deaths would have been expected. The standardised mortality ratio was 1.18 (95% confidence interval 1.02–1.34), indicating a significant increased risk of death in the DCM cohort. Figure 1 displays the observed (± 95% confidence intervals) and expected survival curves.

**Fig. 2:**
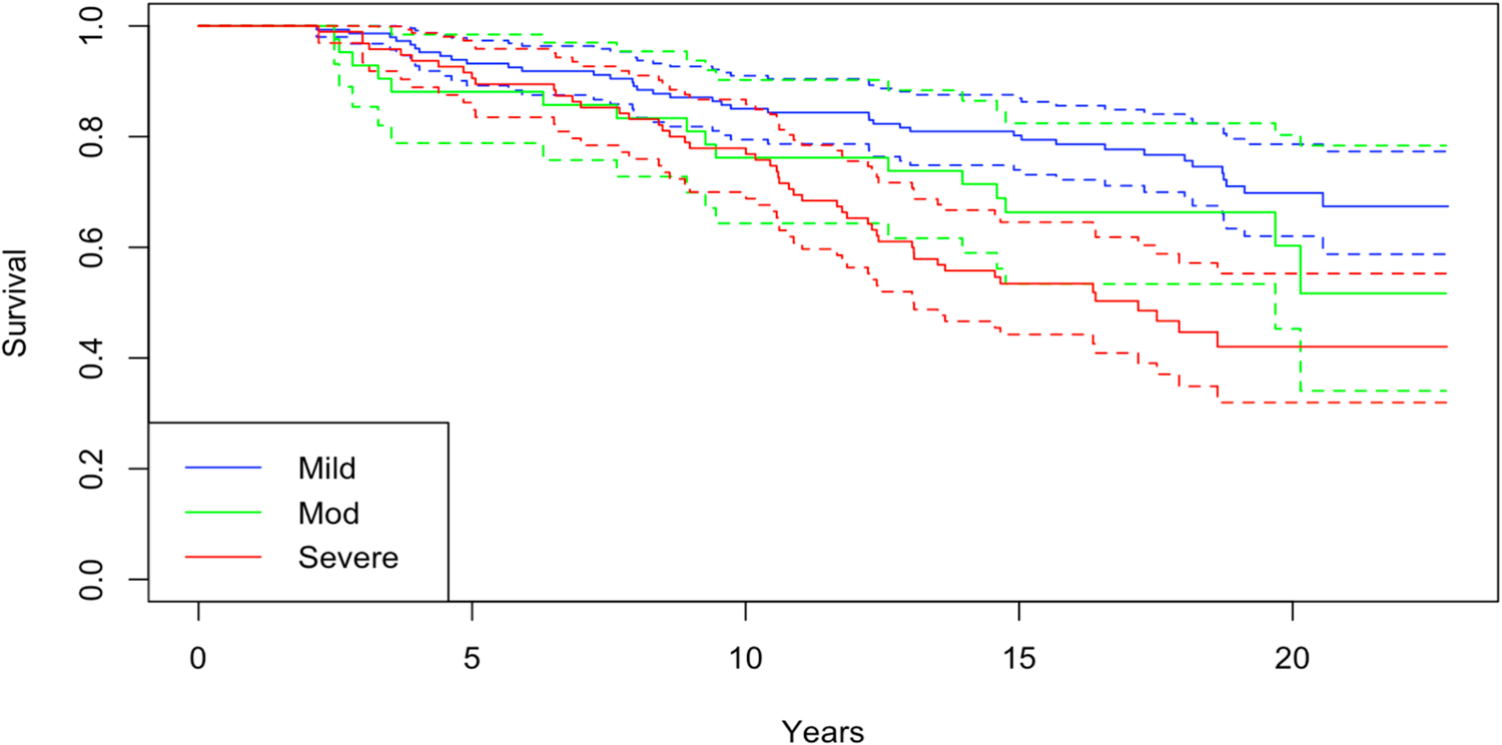
Observed survival based on MDI severity strata. This effect was most marked when dichotimised as severe or not (Supporting Information 2)

Age at operation and disease severity (chi squared 19.5, *p* < 0.001) were associated with observed survival. MCID (chi squared 0, *p* = 0.9) and gender (chi squared 1.9 *p* = 0.2) were not associated with increased survival. MCID and MDI were available for 262 and 287 patients. However, age and gender did not appear to differ between those with and without missing data (Supporting Information 1). Age was modelled across a range of thresholds, shown here in quartiles (chi squared 137, *p* < 0.001) (Fig. 2 and Supporting Information 2) [56].

Variables met the proportional hazard assumption and were entered into a Cox regression model. Backward stepwise elimination was used for variable reduction. Age at operation 1.08 (95% CI: 1.0 to 1.1, *p* < 0.001) and severe DCM 1.6 (95% CI: 1.1 to 2.3, *p* = 0.02) best explained variation in survival (*N* = 287).

**Fig. 3:**
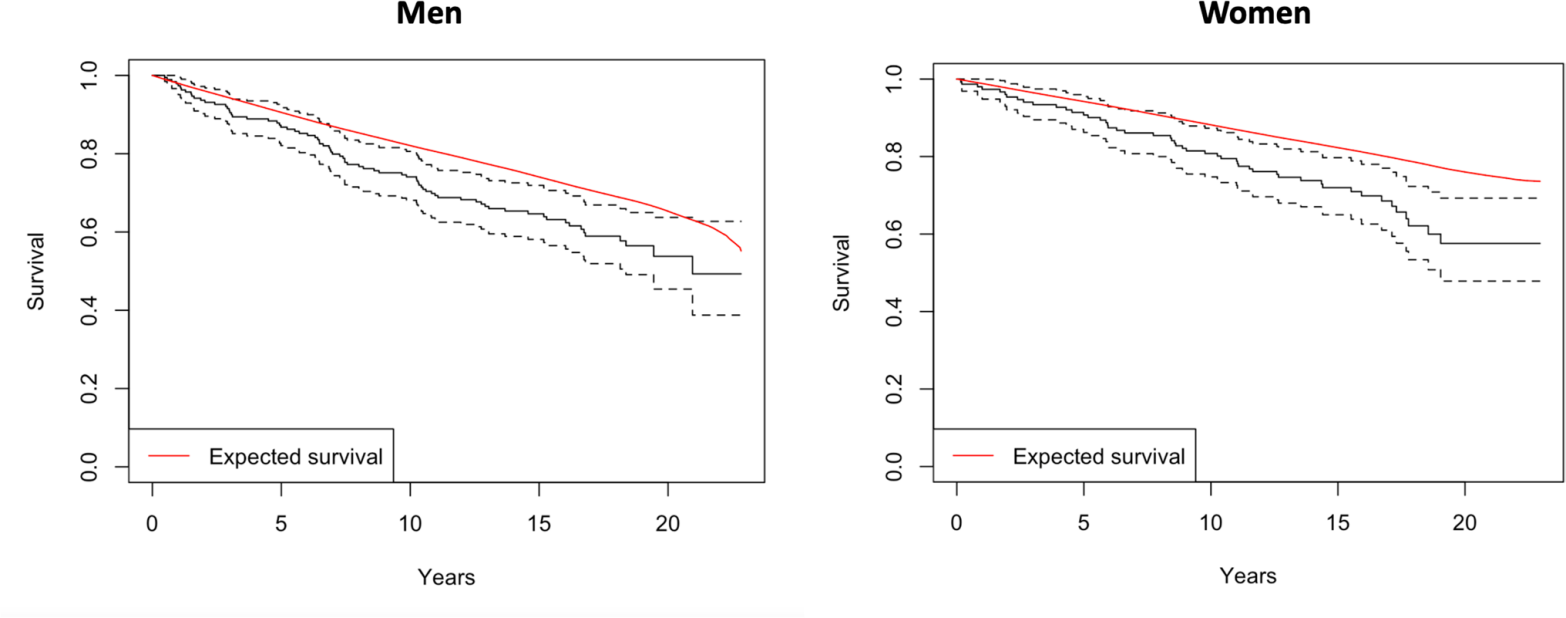
Conditional survival based on MDI

**Fig. 4:**
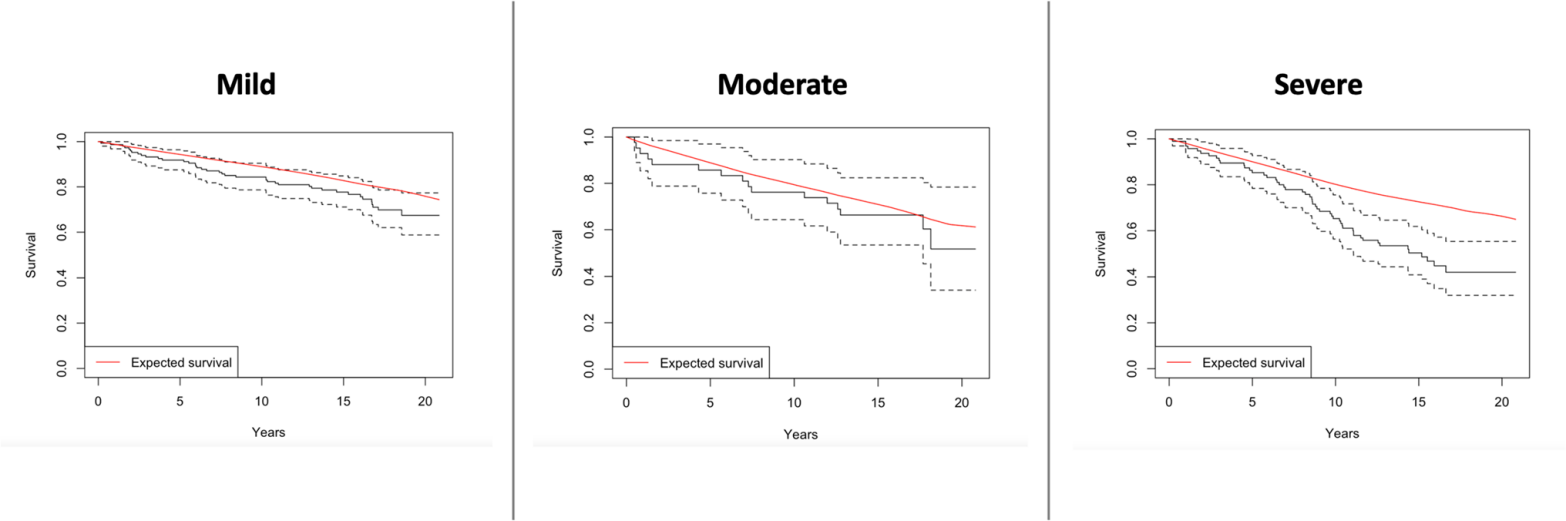
Conditional survival based on gender

For the conditional analysis performed on those surviving 2 years postoperatively, eight patients died within 2 years of surgery. Severe MDI (Fig. 3) and female gender (Fig. 4) had significantly reduced conditional survival. In a Cox regression, only severe DCM was associated with reduced conditional survival (HR 1.6, 95% CI 1.0–2.4, *p* = 0.03).

## Discussion

This is the first study to explore the impact of DCM on life expectancy. It identified, despite surgical treatment, that people with DCM have a reduced life expectancy. Further within multivariate models, across both observed and conditional survival analyses, this was most likely among patients with severe DCM. Age at time of surgery did not predict conditional survival.

There are some important limitations to this analysis. The analysis was restricted to complete datasets, with potentially important covariates for analysis unmeasured in the original study [5, 16], and therefore unable to be included. Furthermore, expected survival was estimated using national survival data, matching on age and gender alone. This could be markedly different to our regional cohort recognised to have above average survival [77] and/or a cohort with disease and co-morbidities such as ischemic heart disease, hypertension, cancer, and others [2, 16]. The exact differences in co-morbidities between the two groups are unknown, which significantly limits the study findings. Further, the exact cause of death was not available for analysis; therefore, deaths related to DCM specifically cannot be delineated. Finally, the findings of this manuscript are generalised to those patients who underwent surgery. Therefore, it is unclear if patients with DCM managed without surgery have a worse survival than the general population.

All patients were identified between 1995 and 2007. While this is important in ensuring adequate follow-up data, this precludes us from measuring the effect of modern treatments on life expectancy, which may further extend DCM survival beyond what is reported in this study. The impact of these treatments will be known in the future.

That said, there is much to confer confidence in this overall finding. Firstly, this represents long-term follow-up from a single surgeon, prospective observational study; the case mix is therefore broadly anticipated to reflect routine practice. Secondly a ‘dose response’ relationship, i.e. that the effect was more pronounced within those with severe as opposed to milder disease, increases the likelihood of a true relationship [37]. Further, this effect was seen both with conditional and observed survival. Finally, and importantly, it aligns with the limited reports from other spinal cord diseases [51, 52]. For example, using a similar approach, Middleton et al. (2012) demonstrated life expectancy was reduced for traumatic spinal cord injury and associated with severity of injury [52].

So, what are the implications for DCM practice and research? Firstly, this finding reinforces the need to consider the long-term consequences of DCM. This is an aspect which has received little research consideration. For example, with respect to surgery, which has been the focus of DCM research to date, most have excluded recurrent disease and considered relatively short-term outcomes [19, 34, 55]. This may be important. For example, many have adopted surgical procedures such as instrumented fusion over decompression alone due to their hypothesised long-term benefits, but these remain unproven [8, 29, 32, 78].

However, more importantly, perhaps, is the indication that this impact would be modifiable. Reduced life expectancy here was driven by those with severe DCM but not observed in those with milder disease. Modelling from surgical outcome data over the last 15 years has demonstrated that disease severity at surgery and length of time with symptoms are critical determinants of outcome [69–71]. As a progressive disease, these factors are likely related [7, 49]. In an updated prediction model, time to treatment within 4 months of symptom onset was predictive of minimal post-operative disability [71]. Put simply, timely treatment could reduce disability in DCM and restore normal life expectancy.

Delivering this however remains a more difficult prospect. Patients today wait, on average, 2 to 5 years for diagnosis, often misdiagnosed and treated for alternative diagnoses first. [10, 28, 41, 62] DCM also often goes undiagnosed [43, 63]; a recent analysis from the UK suggests this could be as much as 90%. [35] Early DCM is also difficult to detect, as DCM can cause a wide variety of symptoms, which may fluctuate, and their frequency or nature in early stages is poorly understood [21, 24, 53, 57]. Further, DCM remains a clinical diagnosis, with MRI only able to support a diagnosis[80], for example, given cord compression is ten times more likely to be incidental [66]. Frontline professionals have received relatively little training on DCM, but even following referral for further investigation, unstructured healthcare pathways and access to MRI mean treatment can still take years [28, 39, 41, 42, 76]. While this remains the greater challenge [27, 43], how and when to offer surgery in mild disease remains a critical knowledge gap. [58, 65] Observational studies have demonstrated that many patients can remain stable for years [3, 9, 64]; consequently, the risks of surgery would seem unwarranted. However, others progress, sometimes quickly. The current guidelines therefore recommend close observation for those managed non-operatively, although exactly what this entails has not been defined. [31] Global research activity is starting to target this knowledge gap, aiming to help stratification through improved diagnostics such as advanced imaging or biomarkers [50], as well as detect disease progression through improved monitoring tools. [72]

The importance of enabling both timely treatment and understanding the long-term implications of DCM and its natural history have been identified as critical research priorities by AO Spine RECODE DCM, a global initiative working to accelerate knowledge discovery in DCM[27]. This process has also formed a minimum dataset, which identified ‘death’ as a core outcome to be measured going forward [22].

The findings of this study therefore reinforce the importance of tackling unexplored aspects of DCM, but in particular, that timely treatment may not just mitigate disability but also save life.

## Conclusions

Life expectancy for people with DCM is reduced despite surgical treatment. This is driven by premature mortality among those left with severe disability. Disability in DCM can be reduced with timely diagnosis and treatment, but many multi-disciplinary and system challenges need to be overcome. These findings therefore reinforce the need for collective and global action [45].

## Supplementary Information

Below is the link to the electronic supplementary material.

Supplementary file1 (DOCX 110 KB)

Supplementary file2 (DOCX 866 KB)

## Data Availability

The original data is available on request.
