# Supplementary material for: Life expectancy in patients with degenerative cervical myelopathy is currently reduced but can be restored with timely treatment
Source: Acta Neurochir (Wien). 2023 Mar 1;165(5):1133–40. doi: 10.1007/s00701-023-05515-8 (PMC10140127; doi:10.1007/s00701-023-05515-8)
Supplement: Supplementary file 1 — Supplementary file1 (DOCX 110 KB) [file 701_2023_5515_MOESM1_ESM.docx]

# Supporting Information 1

Supporting Information 1 : Missing data analysis

|  | |
| --- | --- |
| **** | |
|  | |
| **MCID** | **MDI** |
| *Operation Age*  T Test: p=0.21 | *Operation Age*  T Test: p=0.49 |
| *Gender*  Chi Squared Test: 0.8, p=0.37 | *Gender*  Chi Squared Test: 2.2, 0.34 |
