# Supplementary material for: Life expectancy in patients with degenerative cervical myelopathy is currently reduced but can be restored with timely treatment
Source: Acta Neurochir (Wien). 2023 Mar 1;165(5):1133–40. doi: 10.1007/s00701-023-05515-8 (PMC10140127; doi:10.1007/s00701-023-05515-8)

Supporting Information 2: Observed survival curves for MDI and Age

Exploration of observed survival with disease severity (A) and age, by quartiles (B)


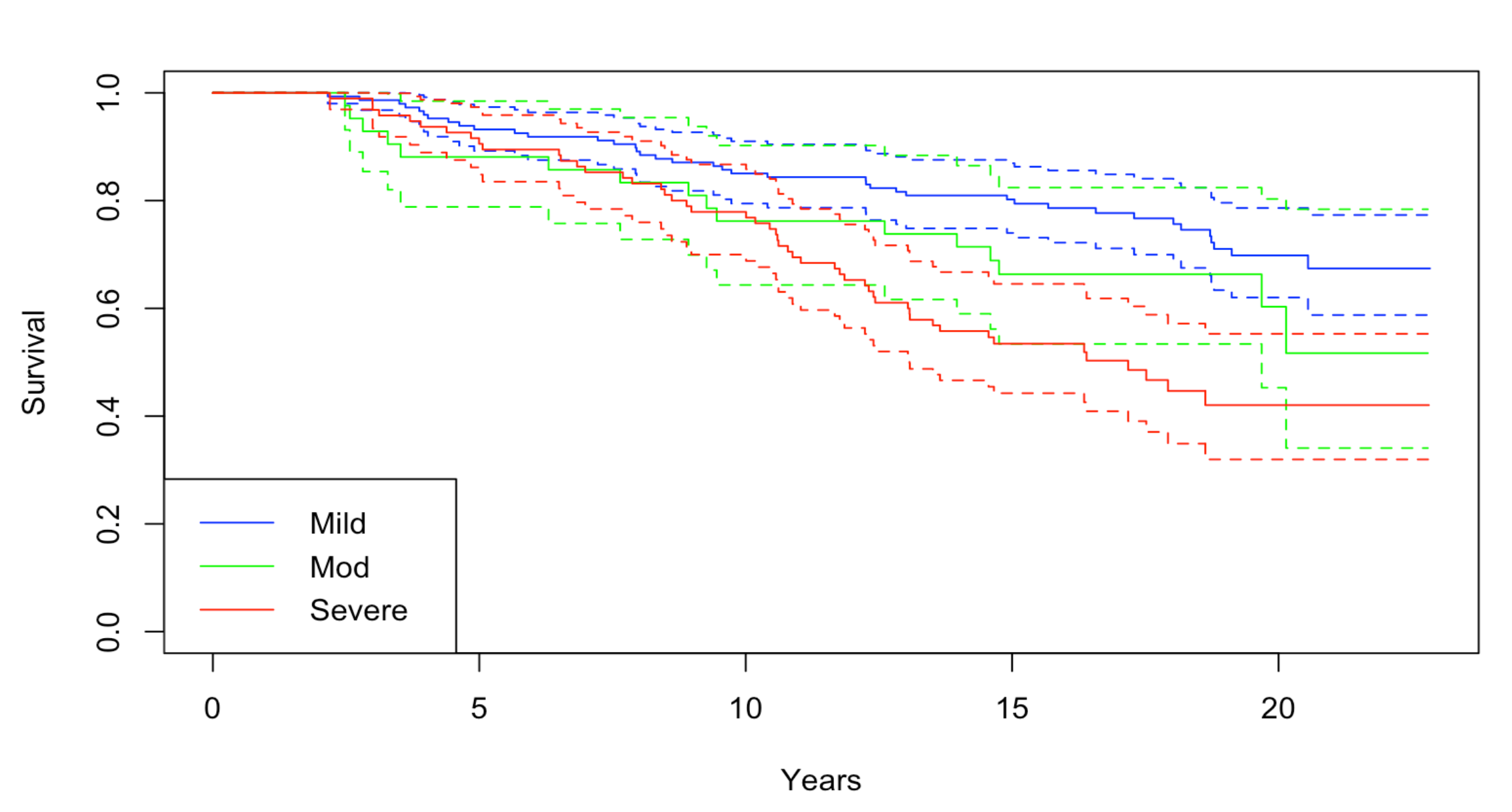


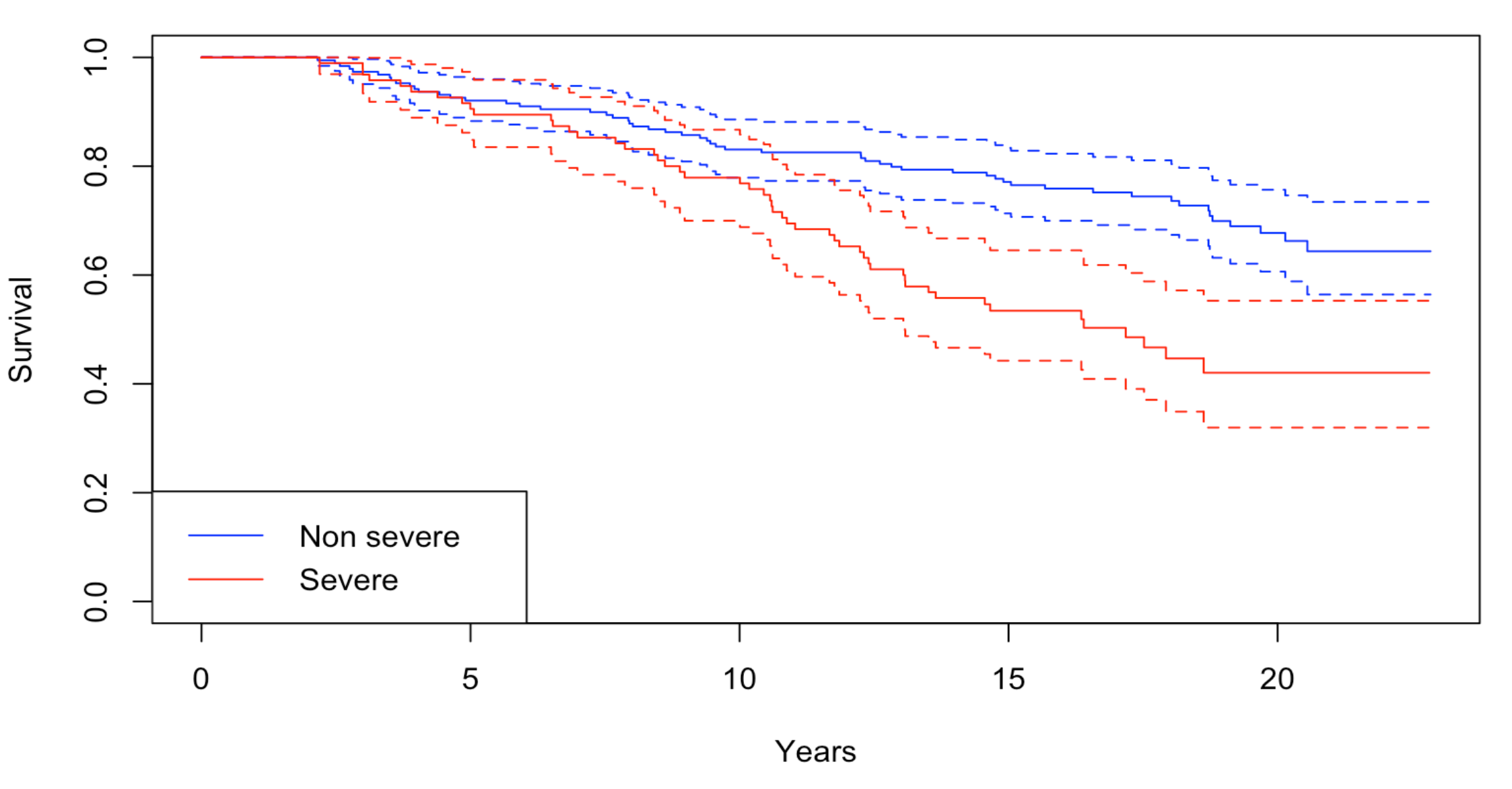


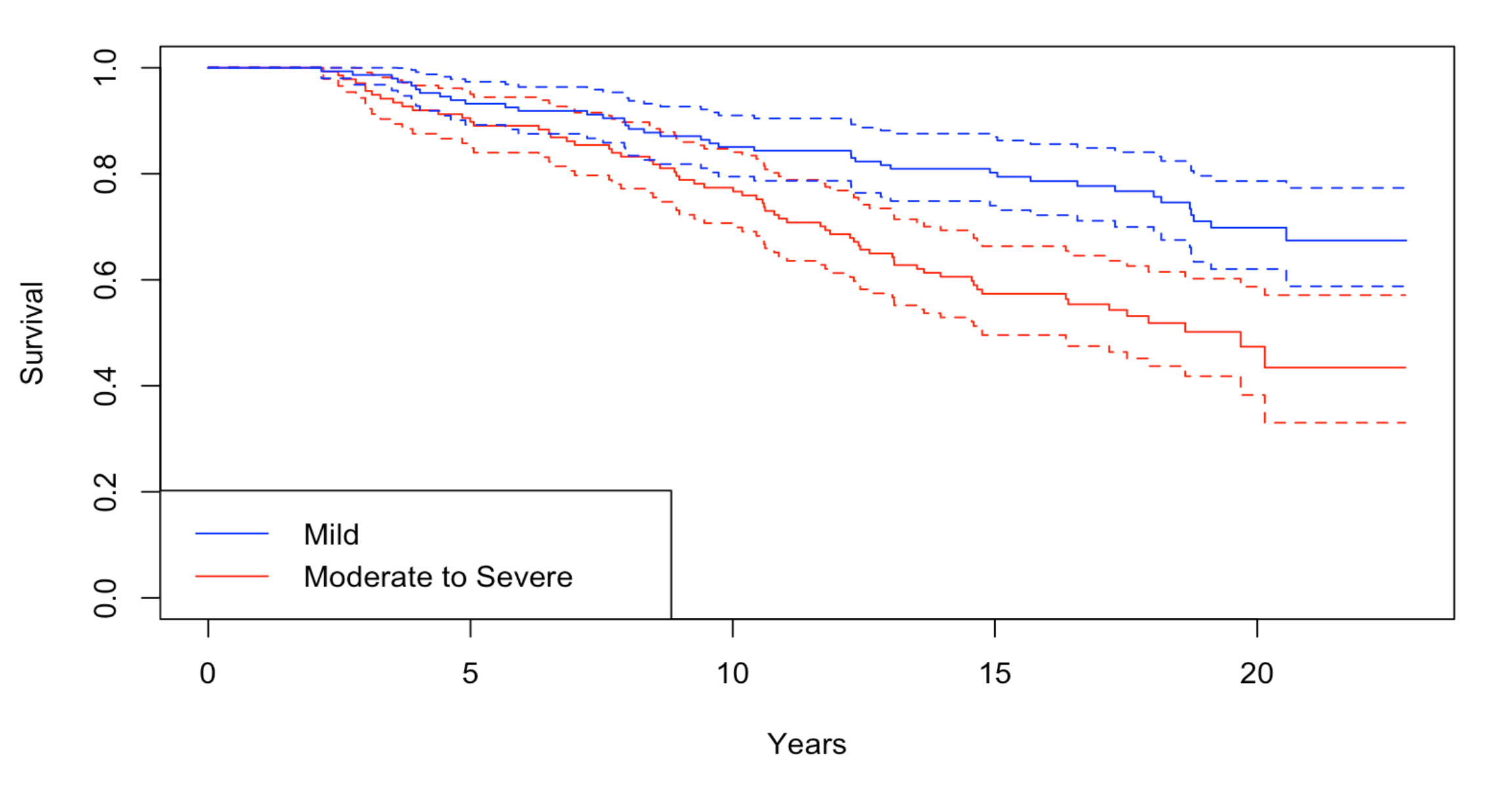


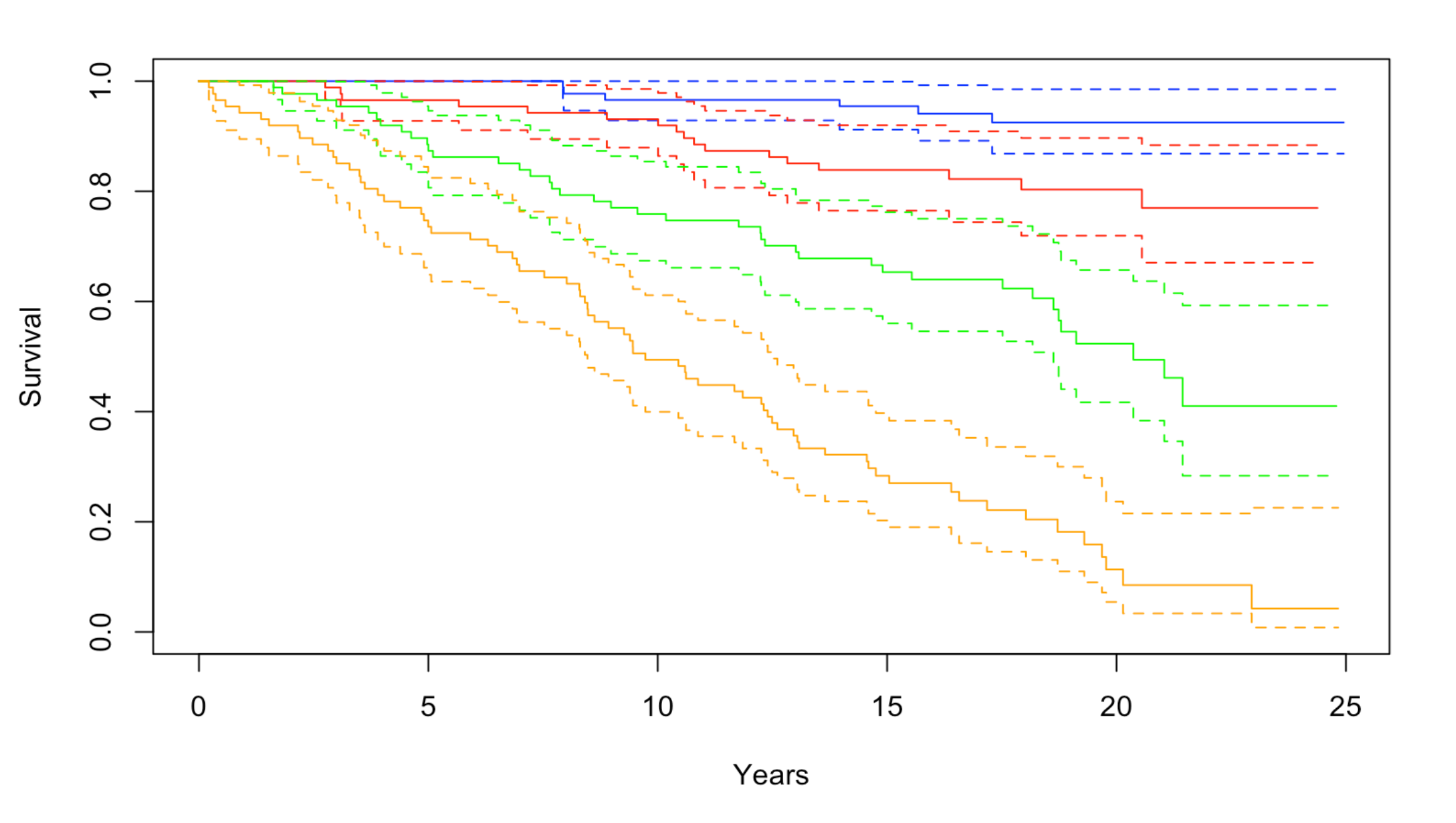

Supplement: Supplementary file 2 — Supplementary file2 (DOCX 866 KB) [file 701_2023_5515_MOESM2_ESM.docx]
